# Gut microbiota as the key controllers of “healthy” aging of elderly people

**DOI:** 10.1186/s12979-020-00213-w

**Published:** 2021-01-05

**Authors:** Emeline Ragonnaud, Arya Biragyn

**Affiliations:** Immunoregulation Section, Laboratory of Immunology and Molecular Biology, National Institute on Aging, 251 Bayview Blvd, Suite 100, Baltimore, MD 21224 USA

**Keywords:** Commensals, IgA, B cells, Aging

## Abstract

Extrinsic factors, such as lifestyle and diet, are shown to be essential in the control of human healthy aging, and thus, longevity. They do so by targeting at least in part the gut microbiome, a collection of commensal microorganisms (microbiota), which colonize the intestinal tract starting after birth, and is established by the age of three. The composition and abundance of individual microbiota appears to continue to change until adulthood, presumably reflecting lifestyle and geographic, racial, and individual differences. Although most of these changes appear to be harmless, a major shift in their composition in the gut (dysbiosis) can trigger harmful local and systemic inflammation. Recent reports indicate that dysbiosis is increased in aging and that the gut microbiota of elderly people is enriched in pro-inflammatory commensals at the expense of beneficial microbes. The clinical consequence of this change remains confusing due to contradictory reports and a high degree of variability of human microbiota and methodologies used. Here, we present the authors’ thoughts that underscore dysbiosis as a primary cause of aging-associated morbidities, and thus, premature death of elderly people. We provide evidence that the dysbiosis triggers a chain of pathological and inflammatory events. Examples include alteration of levels of microbiota-affected metabolites, impaired function and integrity of the gastrointestinal tract, and increased gut leakiness. All of these enhance systemic inflammation, which when associated with aging is termed inflammaging, and result in consequent aging-associated pathologies.

## Introduction

Today, modern humans live markedly longer than their predecessors in the early twentieth century, due to the achievements of modern medicine and lifestyle improvement. The importance of external factors in delaying intrinsic causes of aging-associated pathologies and diseases, i.e., in uncoupling chronological age from physical decline, has been recognized since ancient times. The first prescription to reduce the problems associated with aging, such as dizziness, eye inflammation and ear pain, using a diet of gruel, raw honey, vegetable and fowl is recorded in the book *Hygiene, written by* the Greek physician Galen in 175 AD [1]. Then over century ago, Elie Metchnikoff postulated that senility is caused by “putrefactive bacterial autotoxins” leaked from the colon, and advocated a diet of fermented milk and a “simple” lifestyle to neutralize these autotoxins [2], for the first time emphasizing the importance of gut microbiota in human health and aging. The human gut microbiota is a commensal microbial “superorganism” that consists of trillions of *Bacteria*, *Archaea, Eukarya* and viruses, where four bacterial phyla (*Firmicutes*, *Bacteroides*, *Proteobacteria* and *Actinobacteria*) account for about 98% of the microorganisms. This superorganism coevolved with the host to provide numerous essential and mutually beneficial functions. It supports digestion and absorption of food, metabolizes fibers into bioactive short chain fatty acids (SCFAs), generates vitamins and nutrients, maintains the intestinal integrity, regulates host immunity and directly and indirectly protects from pathogens [3]. The gut microbiota is considered to be a master regulator of immune homeostasis [4], as its absence in germ-free (GF) mice impairs development and maturation of the immune system, while its presence in the gut induces IL-10 and TGFβ-producing Tregs, immunoglobulin A (IgA)-secreting B cells, Th17 cells and type-2 lymphoid innate cells (ILC2) [5–9]. Oral supplementation of mice with 11 commensal strains of bacteria isolated from healthy human feces enhances the host resistance to *Listeria monocytogenes* infection and improves the therapeutic efficacy of immune checkpoint inhibitors in tumor-bearing mice through upregulation of IFNγ-expressing CD8^+^ T cells in the intestine in a CD103^+^ dendritic cell and MHC-I molecule-dependent manner [10]. The microbiota also supports maintenance of the intestinal mucus layer and induces production of various factors and secretory IgA from B cells to promote its own growth and to suppress pathogens.

**Fig. 1:**
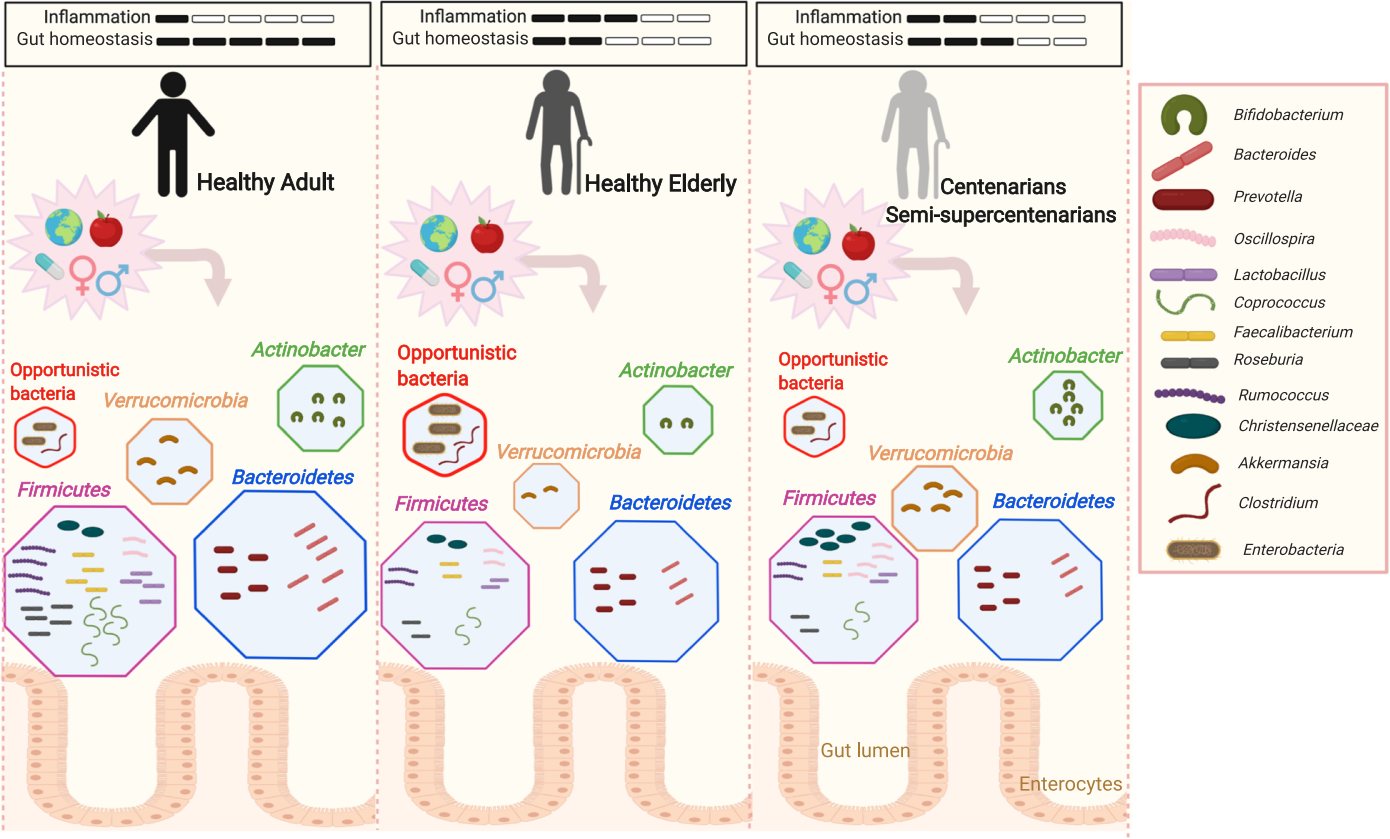
The composition of gut microbiota determines inflammation and possibly lifespan of elderly people. The lumen and particularly the mucin layer of the intestine of young adults are colonized by a diverse population of commensal microbes that co-exist with the host in a symbiotic relationship. Members of *Verrucomicrobia* phylum, particularly *Akkermansia muciniphila,* support gut barrier integrity and thus prevent leakage and subsequent induction of inflammation. In elderly people, the composition of the gut commensals is changed and microbial diversity is reduced due to accumulation of potentially pro-inflammatory commensals and decrease of beneficial microbes, such as members of *Verrucomicrobia*. It therefore leads to gut leakiness and consequent systemic inflammation that facilitates aging-associated morbidities and premature death. Although the microbiota of centenarians changes, its diversity and beneficial commensals are retained, thereby controlling overt inflammation and supporting healthy aging

**Fig. 2:**
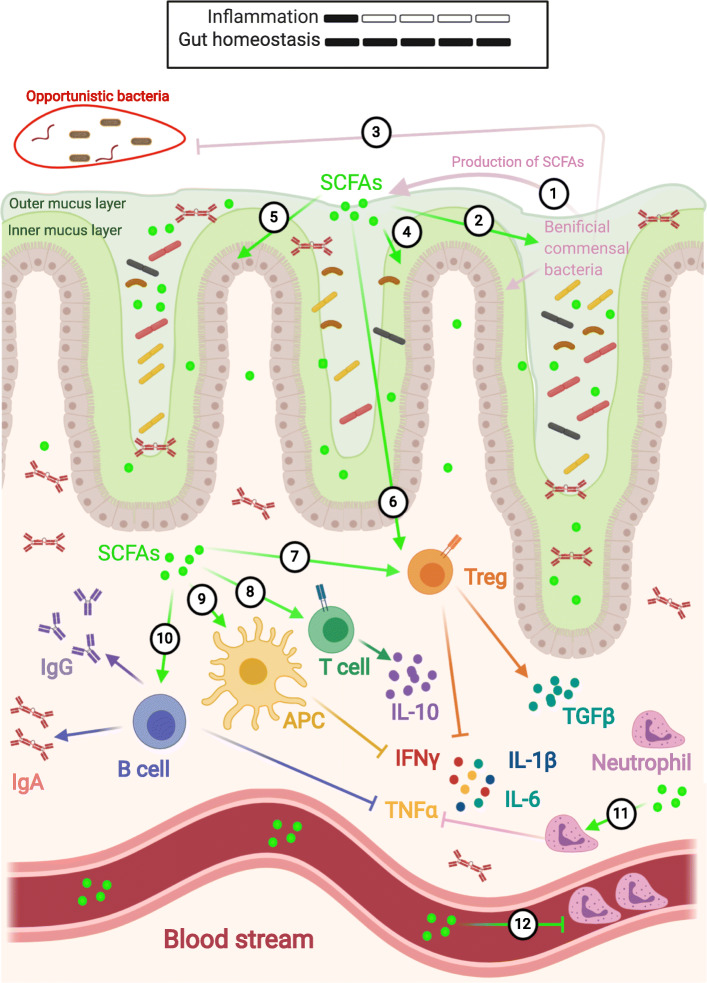
Commensal microbes are sole producers of beneficial SCFAs. 1, In the gut of healthy young people, beneficial commensal bacterial members of Firmicutes produce SCFAs. 2, SCFAs provide energy to the microbiota and 3, thereby can inhibit the colonization of opportunistic bacteria; and 4, promote the production of protective mucus. 5, SCFAs are also source of energy for enterocytes and 6, immune cells. They can cross the gut epithelium layer to promote immune tolerance by 7, inducing TGFβ producing FoxP3^+^ regulatory T cells (Tregs)^,^ 8, IL-10 producing T cells; 9, activating anti-inflammatory responses in antigen presenting cells (APCs); and 10, promoting the production of IgA and IgG from B cells. 11, SCFAs also initiate anti-inflammatory responses in neutrophils and 12, affect their recruitment

**Fig. 3:**
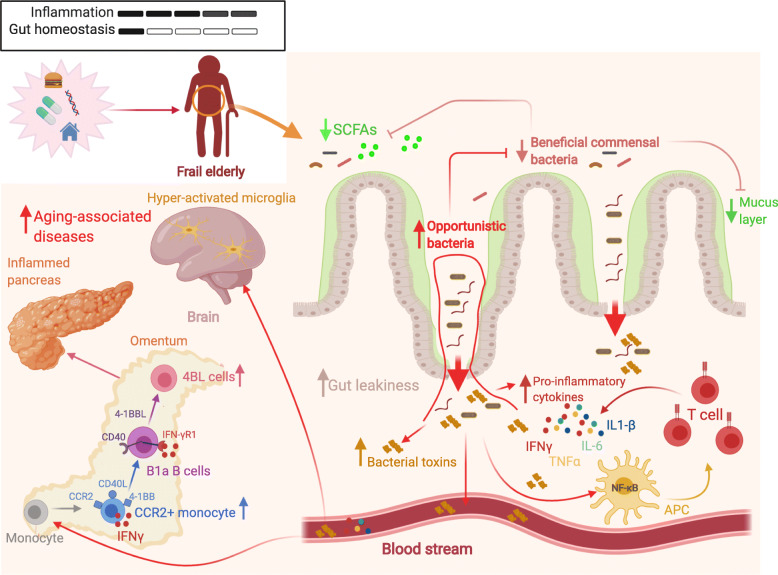
Gut dysbiosis in the elderly increases risk of aging-associated diseases. The composition of the gut microbiota changes with age, causing a mild inflammation in the elderly. This change can be exacerbated by additional intrinsic and extrinsic factors, such as uptake of antibiotics and diet. Frail elderly people show increased gut dysbiosis, a severe decrease of beneficial commensal bacteria, such as *Akkermansia muciniphila* and SCFA-producing bacteria, and a marked increase of opportunistic and potentially proinflammatory commensal microbes. It leads to impairment of the intestinal epithelial integrity and increases gut leakiness and translocation of opportunistic bacteria and endotoxin into the circulation, causing a chain of inflammatory events that enhance the risk of developing aging-associated pathologies. For instance, in aged mice, the *A. muciniphila* loss-caused inflammation recruits and activates CCR2^+^ monocytes in the omentum, where they upregulate 4-1BB, CD40L and the production of IFNγ and convert B1a B cells into 4BL cells via 4-1BBL/4-1BB axis. The 4BL cells then promote insulin resistance in aged hosts

Understanding the role of gut microbiota in human health is hampered by a high degree of variability. Microbial composition differs depending on the conditions within the gastrointestinal tract, such as the high acidity of the stomach and the small intestine and the slightly acidic to neutral pH of the colon. Recent reports from sequencing human fecal microbiota revealed that composition of the gastro-intestinal microbes is affected by human inter-individual, racial, geographic and lifestyle differences. Furthermore, the abundance of its member composition changes depending on the physical state of the host. In people with morbid obesity, microbial composition in the gut shifts from providing benefit to causing harmful inflammation through at least in part impairing the intestinal epithelial integrity. Similar microbiota change has been proposed to occur also in aging and is thought to be a cause of various pathologies and diseases, such as frailty, neurodegeneration, insulin resistance and type-2 diabetes (T2D), cancer, cardiovascular disease and Alzheimer’s disease. Despite an explosion of reports that link gut microbiota to health in aging, the field remains poorly understood and appears to be confusing. Here, we briefly summarize findings from others and also from the authors’ group to emphasize the importance of gut microbiota in the healthy aging of humans. Although dysbiosis and consequent inflammation in aging is assumed to be caused by western style diet (see review [11]), we discuss recent results of microbiota sequencing of elderly people from Italy and China, which suggest that the microbiota change could also be intrinsic to aging process. We propose that the change in gut microbiota is a primary cause of aging-associated pathologies and consequent premature death of elderly people. The most compelling evidence was revealed in studies of genetically homogeneous rodents, such as mice aged in the same environment. It showed that natural aging decreases the microbiota diversity and aged mice have diminished bacterial biosynthesis of cobalamin (B12) and biotin (B7), and SOS genes associated with DNA repair and enhanced creatine degradation, which is associated with muscle wasting [12–14]. Cross-sectional analyses of human fecal microbiota suggest that the fecal microbial population is also altered in elderly people, as in aged mice. The biological and, possibly, aging phenotype in humans appears to depend on four subpopulations (modules) of the “core” fecal microbiota [15]. Although it is difficult to link any given microbe to a clinical outcome, as it may require a cooperative action of multiple microbial species, we also discuss recent findings that underscore the importance of *Akkermansia muciniphila* in healthy aging. *A. muciniphila* is a bacterium that degrades mucin and provides energy to other beneficial microbes, including SCFA-producing bacteria. It also protects intestinal epithelial integrity via activation of epithelial cells and production of mucus, thus supporting housing of beneficial commensals. Its decrease in the gut of aged mice and, possibly, elderly people leads to gut leakiness and consequent induction of a low-level of systemic inflammation in aging (termed “inflammaging”). This presumably explains why increased levels of proinflammatory cytokines in the circulation are associated with an overall loss of fitness and poor health in the elderly [16, 17]. However, the question remains unresolved whether the loss of Akkermansia causes or is caused by the decrease of microbiota diversity, which also occurs in aging and associates with frailty in elderly people [18]. The gut dysbiosis and the loss of beneficial commensals appear to facilitate premature death of elderly people, as fecal microbiota diversity and abundance of *A. muciniphila* are increased in human centenarians [19].

### Does the gut microbiota change with age?

The gut microbiome is an endogenous ecosystem populated with *Bacteria*, *Archaea, Eukarya* and viruses, where four bacterial phyla of *Firmicutes*, *Bacteroides*, *Proteobacteria* and *Actinobacteria* account for 98% of microorganisms. It co-evolved as a symbiotic superorganism with the host to regulate the normal functions of the gut, such as food digestion and absorption of nutrients, and to provide essential vitamins, nutrients, and polyamines. The microbiota degrades undigestible fibers and is thereby exclusively responsible for production of short-chain fatty acids (SCFAs) [20]. SCFAs are involved in multiple processes, such as providing an energy source for microbes and colonocytes, controlling microbial functions, combating pathogens, protecting and maintaining intestinal integrity, and regulating immune cells [3], including differentiation of CD4^+^ T cells and activation of CD8^+^ T cells [21–24]. As such, the absence of microbiota, e.g. SCFA-generating bacteria, in gnotobiotic (germ-free, GF) mice impairs nutrient absorption, dysregulates intestinal morphology, reduces differentiation and maturation of intestinal immune cells such as intraepithelial lymphocytes, Th17 cells, and regulatory T cells (Tregs). This causes shifts in immune responses towards Th2-type cytokines and impairs production of antimicrobial peptides and IgA. The gut microbiota is considered to be a master regulator of immune homeostasis [4], as it induces IL-10 and TGFβ-producing regulatory T cells (Tregs), Th17 cells, ILC2 and IgA-secreting B cells [5–9]. For instance, members of *Bacteroides fragilis* and *Clostridium* strains induce FoxP3^+^ Treg differentiation and production of IL-10 and TGFβ, resulting in the inhibition of inflammation. The intestinal segmented filamentous bacteria (SFB), *Citrobacter rodentium* and fungi like *Candida albicans* induce Th17 cell differentiation and recruitment of neutrophils and other immune cells in the lamina propria (LP), facilitating pathogen clearance.

The microbial colonization of the GI tract starts after the birth of infants by acquiring a few microbial types dominated by the *Bifidobacterium* genus [25]. The *Bifidobacterium* genus dominance declines after the first year of infancy as colonization with other microbes and their diversity increase [25]. The infant microbiota is unstable, and its microbial colonization process is influenced by the mode of delivery and nursing, medications, genetic background [26–33], age, and geographic/cultural traditions [25]. Breast-fed infants promote *Bifidobacterium-*dominated commensals with limited diversity primarily due to human milk oligosaccharides (HMOs), which *Bifidobacterium* species utilize and convert into lactate and short chain fatty acid acetate. In the absence of HMOs, the gut of formula-fed babies is colonized by a diverse population of microbes. The unstable state of the microbiota in infants presumably explains why the gut microbiota of US infants differs from that of non-US ones, as it is dominated with *Prevotella* genus including 28 Operational Taxonomic Units (OTU) [25]. This genus of mucin-degraders is also overrepresented in the fecal microbiota of children from Burkina Faso compared with children living in Italy [34]. The gut microbiota stabilizes when children start eating “solid food” at around 3 years of age and becomes progressively diverse [25, 26, 33, 35, 36]. Fecal microbiome analyses of children and adults of the Amazonas of Venezuela, rural Malawi, and US metropolitan areas indicated that the phylogenic composition and the functional maturation of bacterial communities shift towards adult-like configuration during the first 3 years of childhood [25]. After that age, the gut appears to be protected from microbial colonization, including from supplementation with a cocktail of 11 strains of *Bifidobacterium* and *Lactobacillus* [37], in contrast to having life-long effects from the diet [34, 38].

The gut microbiota of adult humans is dominated by the *Firmicutes* and *Bacteroidetes* phyla and smaller proportions of *Actinobacteria*, *Proteobacteria*, and *Verrucomicrobia* [39]. Sequencing analyses of human fecal metagenomes from four countries identified well-defined and robust microbial communities (termed enterotypes) represented by different levels of three genera: *Bacteroides*, *Prevotella* and *Ruminococcus* [40]. The three enterotypes do not correlate with gender, nationality, body mass index or age. However, the existence of these robust enterotypes remains debatable and instead the diet is considered to be a major driver of microbial composition and function [38, 41] (also see commentary by Jeffery et al., [42]). Moreover, the enterotypes have not been identified in a large study of healthy and frail elderly people [13]. Recently, the study of Italian centenarians (99–104 old), and semi-supercentenarian (105–109 old) suggested the presence of a different core microbiota dominated with *Ruminococcaceae*, *Lachnospiraceae* and *Bacteridaceae* families, which decrease with aging [19]. An independent study of Chinese centenarians revealed a negative association between extreme aging and the abundance of *Coprococcus*, *Roseburia* and *Faecalibacterium* genera, belonging to the *Lachnospiraceae* and *Ruminococcaceae* families [43, 44]. Despite differences in race, lifestyle and diet of the centenarians, the two studies found 11 shared features among the top 50 microbes, such as comparable changes in members of *Blautia*, *Clostridium* cluster XIVa, *Faecalibacterium*, *Escherichia_Shigella*, unclassified Lachnospiraceae, Ruminococcaceae, and Erysipelotrichaceae. Other groups reported that the enrichment of *Bacteroidetes* and *Protobacteria* abundances and decrease in species of *Bifidobacteria* and *Lactobacilli* in aged people [18, 45–50]. Interestingly, the Italian and Chinese study of centenarians found longevity increases microbial community richness (Chao index and observed operational taxonomic units, OTUs) and the abundance of subdominant but health-related bacterial genera and families, such as *Oscillospira, Christensenellaceae, Akkermansia* and *Bifidobacterium* [19, 44]. This presumably implies a healthy state of the elderly cohorts because *Oscillospira* and *Christensenellaceae* control leanness and decrease certain inflammatory diseases in humans [51, 52]. *A. muciniphila* protects the intestinal epithelial integrity, supports beneficial SCFA-producing bacteria and reduces inflammation and metabolic impairments such as insulin resistance [53, 54]. *Bifidobacterium* generates lactate and SCFA acid acetate and reduces pro-inflammatory microbes [55]. The aging-associated increase of *Oscillospira,* when compared to children and middle-aged adults, was also noted in recent multivariant unsupervised reanalysis of 16S DNA sequencing data from 371 people ranging from newborn babies to centenarians [56].

Overall, despite significant interindividual variability and influence of external factors such as diet, medications, type of exercise or mobility, and the geographical locations of the host [13, 25], the composition of the gut microbiota changes progressively as people age [56]. Although the health status of subjects is often not reported, we can conclude that unlike young and middle-aged adults, the gut of elderly people is reduced in beneficial and enriched in pro-inflammatory commensal microbes. This change is presumably “intrinsic” to the aging process, as it also occurs in genetically homogeneous mice aged and fed in the same condition. Aged mice exhibit a decrease in beneficial gut bacteria, such as *A. muciniphila* and SCFA-producers in *Clostridium* members of cluster IV, and an increase pro-inflammatory microbes [53]. The changes in the gut microbiota composition with age are illustrated in Fig. 1. 

### Does the alteration of the gut microbiota affect the health of elderly people?

Metchnikoff’s original concept published over a century ago explains the disabilities of elderly people by accumulation of “putrefactive bacterial autotoxins”, which leaked from their colon. He postulated that this leakage results in the conversion of phagocytes into destroyers of healthy tissues [2]. This idea was confirmed centuries later by demonstrating the importance of chronic inflammation on activation as well dysfunction of phagocytic cells. Fecal microbiota markedly differs in people living in community-dwelling and long-term care nursing facilities [12, 13, 57, 58], consistent with its role in healthy aging. Dysbiosis, increased microbiota instability and loss of its members in community-dwelling adults can diminish numerous benefits of commensal microbes to the host [59]. This probably explains why decrease of the gut microbial diversity escalates as the frailty of elderly humans increases [18, 60–62], positively associating the gut dysbiosis of community-dwelling adults and the biological, but not chronological, age [60, 62]. Fecal microbiota of elderly frail people and aged mice is enriched in the *Bacteroidetes* phylum and the *Oscillibacter* and *Alistipes* genera and *Eubacteriaceae* family and is reduced in *Faecalibacterium* and *Lactobacillus* [12–14, 18]. Similarly, our group reported that elderly macaques and mice acquire increased insulin resistance because of gut dysbiosis, such as marked decrease of beneficial commensals and their metabolite SCFAs in the gut and in the circulation and increase of pro-inflammatory microbes [53]. A primary driver of this process in aged mice was *Akkermansia*, whose decrease caused intestinal leakage and activation of CCR2^+^ monocytes to convert innate B1a B cells into pathogenic 4-1BBL^+^TNF^+^ B cells (termed 4BL cells), which in turn induced insulin resistance via the 4-1BBL/4-1BB axis [53]. Conversely and despite lifestyle and racial differences, both Chinese and Italian centenarians show an increase of the alpha diversity in the top 500 OUTs [19, 44] (although the Simpson reciprocal index of diversity was reduced in Italian centenarians [63]), suggesting that healthy aging may benefit from the retention of richness of commensal microbes in the gut. Overall, the decreased diversity of the intestinal commensal microbes, which often manifests in reduction of beneficial and enrichment of pro-inflammatory members, can have a detrimental consequence in healthy aging and thus longevity.

### The importance of microbial metabolites such as short chain fatty acids in immunity

Although the composition of the gut microbiota changes with age, the core of microbiota does not age per se [18]. Fecal DNA sequencing and PCR analyses mostly reveal change in abundance of its individual members, which as a part of a symbiotic ecosystem then affects the microbial community and their crosstalk with the host. The abundance of *Bifidobacterium-*dominated commensals in the gut microbiota of breast-fed infants, which generates lactate and a beneficial SCFA (acetate) from milk oligosaccharides, progressively decreases together with the diversification of the microbial community after weaning and transition to adulthood [25]. Their decrease in young adults is complemented by other bacteria that metabolizes fibers to SCFAs, such as by their key producer Firmicutes [64, 65]. However, both *Bifidobacterium* and some members of Firmicutes, such as *Clostridium* clusters IV (*Ruminococcus obeum et rel*., *Roseburia intestinalis et rel*., *E. ventriosum et rel*., *E. rectale et rel*., *E. hallii et rel.)* and of *Clostridium* cluster XIVa, (*Papillibacter cinnamovorans et rel*., and *F. prausnitzii et rel*.), are decreased in aging and in centenarians [63]. This explains why the abundance of *Bifidobacterium* inversely correlates with inflammaging in elderly people and centenarians [63, 66], consistent with their benefit in prolonging longevity of mice as inhibitors of pro-inflammatory cytokines (for example from macrophages) and colonic senescence, and inducers of colonic tight junctions and mucus production [55, 67].

SCFAs provide energy to commensal microbes, immune cells and the colonic epithelium, and induce production of mucus e.g., they protect the epithelial barrier functions, support the growth of various beneficial commensal microbes, and promote immune tolerance and gut homeostasis [68]. SCFAs also regulate immune responses by directly stimulating immune cells. In airways of patients with cystic fibrosis, the increase of SCFAs, such as acetate, propionate, and butyrate, associates positively with neutrophil infiltration in the sputum and negatively with expansion of *Pseudomonas aeruginosa*. The SCFAs induce production of IL-8/CXCL8 and release of granulocyte-macrophage colony-stimulating factor (GM-CSF) and granulocyte colony-stimulating factor (G-CSF), which are needed in the recruitment and persistence of neutrophils, while inhibiting the synthesis of nitric oxide synthase involved in airway inflammation [69]. However, the increase of SCFAs, which can be achieved by feeding with a high-fiber diet, inhibits neutrophil recruitment and consequently airway inflammation and thereby enhances survival in influenza-infected mice [22]. In this study, SCFAs also impair infiltration of neutrophils in the lung airways because they recruit bone marrow-derived Ly6c^−^ patrolling monocytes, which upon their differentiation into alternatively activated macrophages reduce expression of neutrophil-recruiting CXCL1 chemokine as well activate effector functions of CD8^+^ T cells. Extracellular SCFAs are also utilized as substrates for β-oxydation of fatty acids in CD8^+^ T cell [70] and colonocyte metabolism [71] after they are taken up by several G-protein coupled receptors (GPRs). Butyrate uncouples the tricarboxylic acid cycle from glycolytic input and enhances memory potential and recall responses of antigen-primed CD8^+^ T cells via GPR41, and GPR43 [70]. By targeting GPR109A [72], GPR41 and GPR43 [73–75], which are expressed on the surface of macrophages, dendritic cells and neutrophils, butyrate induces anti-inflammatory responses [76]. Similarly, through the interaction with GPR109A, it promotes anti-inflammatory pathways in colonic macrophages and dendritic cells and induces differentiation of Tregs and IL-10-producing T cells [77]. Butyrate upregulates expression of transcription factor Blimp-1 and IL-10 production in Th1 CD4^+^ T cells (without affecting conversion of Tregs or Th17 cells) via GPR43 and controls colitis [78]. SCFAs from *Clostridium* species clusters IV and XIV induce and regulate colonic FoxP3^+^ Tregs [79, 80], although other SCFA non-producer microbes also support this process as they are required in the restoration of Tregs in GF mice after butyrate supplementation [9]. Butyrate is a potent inhibitor of histone deacetylases (HDACs), and its internalization via the above-noted GPRs or other unknown pathways causes epigenetic modifications of non-hematopoietic and hematopoietic cells [81–85], promoting anti-inflammatory immune responses through inhibition of the production of multiple pro-inflammatory cytokines (IFN-γ, IL-6, IL1-β) and induction of the release of anti-inflammatory IL-10 and TGF-β [86]. As an inhibitor of HDAC3, butyrate induces differentiation of monocytes to macrophages as well as metabolic and transcriptional changes in macrophages enhancing their bactericidal functions [87]. SCFAs inactivate nuclear factor-κB (NF-κB) and reduce production of TNFα in peripheral blood mononuclear cells and neutrophils [88, 89]. By supporting differentiation of antibody-producing B cells, SCFAs increase both intestinal IgA and systemic IgG responses to combat pathogens [90]. Upon entering the bloodstream and reaching distant organ sites such as brain and lungs, SCFAs control systemic immune responses [91]. By regulating Tregs in the lungs, SCFAs protect against allergic airway diseases [21, 92, 93]. In the brain, SCFAs directly affect microglia maturation and function [94] and thereby reduce neuroinflammation [95]. However, SCFAs can also exert pro-inflammatory responses under certain conditions [69, 96, 97]. For instance, SCFAs can aggravate colitis-associated inflammation in mice by inducing expression of T-bet and IFNγ in Tregs and in conventional CD4^+^ T cells [96]. Also, SCFAs can exacerbate inflammation in airway epithelial cells [69], and the generation of IL-17 and IFNγ producing T cells in the CNS in mice with multiple sclerosis [97]. In the mouse model of Parkinson’s disease (PD), SCFAs are linked to acceleration of pathogenic α-synuclein (αSyn) aggregation and motor deficits [98]. The different functions of SCFAs in the gut microbiota are illusrated in Fig. 2. 

### Alteration of the SCFA levels in elderly people

As discussed above, SCFAs are produced by a number of bacterial populations working in concert with a community of commensals [99]. For example, *A*. *muciniphila* not only supports growth of other commensal microbes by liberating oligosaccharides as well maintaining the mucus layer, but also induces butyrate production from *Anaerostipes caccae, Eubacterium hallii,* and *Faecalibacterium prausnitzii*, while *E. hallii* upregulates pseudovitamin B12 to stimulate propionate production from *A*. *muciniphila* [100]. Butyrate improves barrier function of the intestinal epithelial cells (IECs) and protects them from *C. difficile* toxin damage by activating hypoxia-inducible factor 1α [101]. This probably explains why the gut of centenarians retains high levels of butyrate and has increased quantities of the butyrate producers *Anaerotruncus colihominis et rel*. (from *Clostridium* cluster IV) and *Eubacterium limosum et rel*. (from *Clostridium* cluster XV), *Bifidobacterium* and other the health-associated bacteria, such as *Akkermansia* and *Christensenellaceae* [19, 44, 99]. *A. muciniphila* and its outer membrane protein Amuc_1100* also stimulate IECs to induce mucus production necessary for protection of the intestinal barrier integrity and the support of other beneficial commensals [102–104]. Therefore, the decrease of this important bacterium causes gut dysbiosis and impairs the intestinal epithelial integrity, which increases gut leakiness and systemic endotoxemia. A resulting chronic proinflammatory state with higher levels of circulating IL-6, IFNs, TNFα, and IL-1 then promotes poor fitness and frailty in elderly humans [16, 17, 44, 63, 105]. Our group recently reported that the decrease of *A*. *muciniphila* in the gut of aged mice triggers a chain of inflammatory events that manifests in the increase of insulin resistance [53]. It starts from the reduction of the colonic mucin layer, which presumably leads to the loss of commensal bacteria producing butyrate, such as *I*. *butyriciproducens*, *F*. *prausnitzii*, *R*. *faecis*, and *A*. *butyraticus*, and thus a reduction in SCFAs, particularly butyrate, in the gut lumen and in the circulation. The decrease of this important source of energy to colonocytes and suppressor of proinflammatory and pathogenic commensal bacteria [106] further enhances dysbiosis and gut leakage in aged mice, thereby sustaining inflammaging [53]. Because butyrate is also a potent inhibitor of TLR4 signaling [107], its decrease in the circulation enables bacterial stimuli, such as endotoxin, to freely induce inflammation in the omentum and to upregulate expression of 4-1BB, CD40L, and IFN-γ in BM-derived inflammatory CCR2^+^ monocytes. Upon infiltration into the omentum, these monocytes convert innate B1a B cells into 4BL cells [53] via 4-1BBL, CD40, and IFNγR1 signaling. The 4BL cells then promote insulin resistance in aged hosts, at least in part, utilizing the 4-1BBL/4-1BB axis [53]. A similar pathway appears to exist in elderly humans and macaques, as their increased insulin resistance associates with 4BL cells induced by aging-activated monocytes [53, 108, 109]. In elderly people, the level of SCFAs from carbohydrate fermentation is decreased while metabolites from protein fermentation (branched fatty acids, ammonia and phenols) are increased, indicating a shift from saccharolytic fermentation to unfavorable proteolytic activities [110, 111]. This shift occurs progressively as elderly people age [99], and can be accelerated upon the use of antibiotics or with low-fiber diets [110, 112, 113]. The decrease in the level of SCFAs enhances susceptibility to opportunistic bacterial species and pathogens. For instance, treatment with the antibiotic cefoperazone reduces SCFAs and thereby increases colonization of the fungus *Candida albicans* in mice [114]. Similarly, antibiotic-induced depletion of butyrate-producing microbes decreases butyrate levels, which leads to epithelial oxygenation and expansion of aerobic bacteria such as *Escherichia coli* and *Salmonella enterica* [106, 115]. Mechanistically, the colonocyte oxygenation is primarily linked to decreased activity of the intracellular butyrate sensor peroxisome proliferator-activated receptor γ (PPAR-γ), which leads to activation of iNOS and increase of nitrate levels [106]. The mechamisms in which gut dysbiosis increases age-related diseases are illustrated in Fig. 3.

### Contribution of other microbial factors in inflammaging

The gut microbiota controls local immune responses, such as the development and regulation of the intestinal immune responses [116]. Germ-free rodents show an alteration in the development of the gut-associated lymphoid tissues (GALT) and maturation of isolated lymphoid follicles (ILFs), leading to significantly lower numbers of T cells, including Tregs, and IgA-producing B cells [116, 117]. On the other hand, dysbiosis induces systemic inflammation, explaining relatively high plasma levels of MCP-1/CCL2 [118], IL-8/CXCL8, TNFα, IL-6, and C-reactive protein (CRP) in elderly people living in long-term care facilities [13]. Underscoring the importance of gut microbiota, the inflammatory phenotype in aging is also transferable, as GF mice show signs of inflammaging after transplantation with fecal microbiota of from aged, but not young, mice [119]. Similarly, the fecal transplantation from aged, but not young, mice induces the genration of 4BL cells in GF mice, primarily due to absence of *A. muciniphila* and SCFA-producer bacteria [53]. However, gut microbiota modulates inflammaging through a plethora of additional mechanisms. For instance, the gut of long lived elderly people is decreased in proinflammatory *F. prausnitzii* and is increased about 15-fold in *E. limosum* [63]. Elderly people and centenarians also markedly decrease abundance of *Bifidobacterium* genus in the gut [25, 63, 66], presumably explaining increased inflammaging and consequent aging-associated morbidity and mortality. *Bifidobacterium* is an important producer of lactate, which affects acidity of the environment, and polyamines that scavenge reactive oxygen species (ROS). It also induces stress-response genes, regulates NFκB activation, inhibits pro-inflammatory cytokines from macrophages, suppresses colonic senescence, maintains colonic tight junctions and mucus production, and even prolongs longevity in mice [55, 67], implying that *Bifidobacterium* supplementation may also reverse aging-associated impairments in elderly humans. However, recent systematic reviews and meta-analyses of randomized numerous probiotic trials revealed no significant benefit in protection from infection, improving NK cell function, quality of life and mortality of elderly people as compared with placebo controls [120, 121]. Despite importance of probiotics, the key question remains unresolved – whether they can efficiently colonize the gut of adults and elderly people. Recent study failed to find the colonization evidence in adults supplemented with a cocktail of 11 *Bifidobacterium* and *Lactobacillus* strains failed [37]. It is also unclear whether orally given probiotics can survive acidity of the stomach and competition from the commensal microbiota. Lastly, if the bacterial decrease in aging is caused by the immune system, attempts to restore them, for example by supplementing *Bifidobacterium*, may instead accelerate their loss due to activation of the immune response.

### Concluding remarks

Despite significant interindividual and lifestyle differences, the composition of the gut microbiota of elderly humans markedly differs from that of young and middle-aged adults. The compositional shift coincides with the onset of immune dysregulation and manifestation of aging-associated pathologies, e.g. ≥ 70 years of age. In elderly, particularly frail people, the composition of the gut microbiota shows signs of dysbiosis, such as a marked decrease in diversity of its population due to the accumulation of proinflammatory commensals and reduction of beneficial microbes. The decrease of beneficial microbes, particularly supporters of mucin production and producers of SCFAs, appears to be essential in triggering a chain of inflammatory events, such as the impairment of intestinal barrier integrity and increase of gut leakiness, endotoxemia and subsequent inflammaging and aging-associated morbidities. As in aged fruit flies where intestinal permeability increases mortality [122, 123], we therefore propose that the gut dysbiosis and leakiness is a major cause of premature death in elderly people. Consistent with ancient philosophers and Elie Metchnikoff's vision and as lifestyle improvements show, extrinsic manipulations can control the ills of the elderly to maintain healthy aging. This concept is validated in modeling studies in rodents, revealing that aging-associated pathologies are reversible. For example, the gut microbiota-induced inflammaging and consequent increased insulin resistance is reversed by restoring *A. muciniphila,* supplementing with butyrate, or inactivating 4BL cells and monocytes in aged mice and macaques [53]. On the other hand, little is known about the cause(−s) of dysbiosis, the safety and potential health risk of microbiota-based interventions in elderly people. Further research is needed to fully understand the benefit of probiotics and their use in humans.

## Data Availability

Yes
